# Faunistic study of butterflies (Lepidoptera, Papilionoidea) of Sulaymaniyah Province, Kurdistan-Iraq

**DOI:** 10.3897/BDJ.10.e82612

**Published:** 2022-03-25

**Authors:** Farhad A. Khudhur

**Affiliations:** 1 University of Sulaimani, Sulaymaniyah, Kurdistan Region, Iraq University of Sulaimani Sulaymaniyah, Kurdistan Region Iraq; 2 University of Mendel, Brno, Czech Republic University of Mendel Brno Czech Republic

**Keywords:** butterflies, Papilionoidea, Lepidoptera, Sulaymaniyah, Iraqi fauna

## Abstract

**Background:**

This study investigates the butterfly fauna (Lepidoptera, Papilionoidea) of Sulaymaniyah Province, in Kurdistan Region, Iraq. Investigations were carried out between April 2016 and April 2021, during which butterfly specimens were collected from 34 different localities throughout Sulaymaniyah Province. The collected butterflies belonged to 103 species within five families: five species of Papilionidae, 19 species of Hesperiidae, 18 species of Pieridae, 25 species of Lycaenidae and 36 species of Nymphalidae.

**New information:**

Eight species, *Carcharodusstauderi* Reverdin, 1913, *Thymelicushyrax* Lederer, 1861, *Gonepteryxrhamni* (Linnaeus, 1758) *Pieriskrueperi* Staudinger, 1860, *Coliaserate* Esper, 1803, *Polyommatusthersites* (Cantener, 1835), *Brenthismofidii* Wyatt, 1968 and *Pseudochazaramamurra* Herrich-Schäffer, 1852 have been added as new records to the fauna of Iraqi butterflies.

## Introduction

Sulaymaniyah Province is a mountainous area located in the northeast of Iraq and the southeast part of the Iraqi Kurdistan Region. As a part of the Iraqi Kurdistan Region biogeographically, Sulaymaniyah is situated in the Irano-Tauranian in the southeast of the western Palearctic realm (Ararat 2009). The Province mostly lies between 34°50′ to 36°50′ N longitude and 44°70′ to 46°00′ E latitude, elevation ranges 150-2550 m a.s.l. Sulaymaniyah has a diverse topography of mountains, hills, plains, valleys and lakes, with great biodiversity and many diverse habitats (Ararat 2009, Ahmed 2016).

Butterflies belong to a single superfamily, Papilionoidea (Heikkilä et al. 2011, Wiemers and Verovnik 2016). This superfamily consists of seven families; Papilionidae, Hedylidae, Hesperiidae, Pieridae, Riodinidae, Lycaenidae and Nymphalidae (van Nieukerken et al. 2011, Wiemers and Verovnik 2016). In the past, the Lepidopteran faunal compositions have been rarely studied in Iraq in general and, particularly, in Sulaymaniyah. Butterfly recording in Iraq seemed to begin with Peile (1921), who published his notes on 44 species of the butterfly taken from Mesopotamia and another 44 species collected from adjacent highlands of northwest Persia and South Kurdistan. Scott (1929), in his entomological journey to the Sulaymaniyah Province, failed to record any butterfly species. The first and major contribution to the Lepidoptera fauna of Iraq was published by the British Colonel Wiltshire (1957). This work was a compilation of his intensively published articles (Wiltshire 1937a, Wiltshire 1937b, Wiltshire 1939a, Wiltshire 1939b, Wiltshire 1941, Wiltshire 1944). One year later, Higgins (1958), during his visit to Kurdistan, listed notes about 65 species of butterfly and added several new records to the known butterfly fauna of Iraq. During the 1970s, only a single Nymphalid species was recorded in Iraq (Igarashi 1971). There are several other published works considering Lepidoptera fauna in Iraq, which mostly depend on literature linked to Iraqi butterfly fauna (Kemal and Koçak 2011, Kemal and Koçak 2018, Koçak and Kemal 2001). Colonel Wiltshire’s data have been used to map Iraqi butterflies by Tshikolovets et al. (2014) in a book entitled “The butterflies of Iran and Iraq”, in which they studied also some fragmentary data from literature linked to Iraqi butterfly fauna and a few museum specimens. Recently in 2018, three articles have been published dealing with the butterfly fauna of Iraqi Kurdistan. These three studies have added several species to the known Iraqi fauna in the form of new records (Othman et al. 2018, Said et al. 2018, Yakovlev 2018), fifty-five species in the Erbil Province (Othman et al. 2018) and fifty species in the Duhok Province (Said et al. 2018, Yakovlev 2018). Lately, two lycaeniid butterfly species of the genus *Polyommatus* have been added to the Iraqi's butterfly fauna (Khudhur 2021). Based on literature data, the total recorded number of butterfly species in Iraq is 154 (Wiltshire 1957, Higgins 1958, Igarashi 1971, Othman et al. 2018, Said et al. 2018, Yakovlev 2018, Khudhur 2021).

The current study aims to investigate the butterfly fauna in the Sulaymaniyah Province and to contribute to the knowledge of Papilionoidea species and their distribution in Iraq.

## Materials and methods

**Figure 1.**
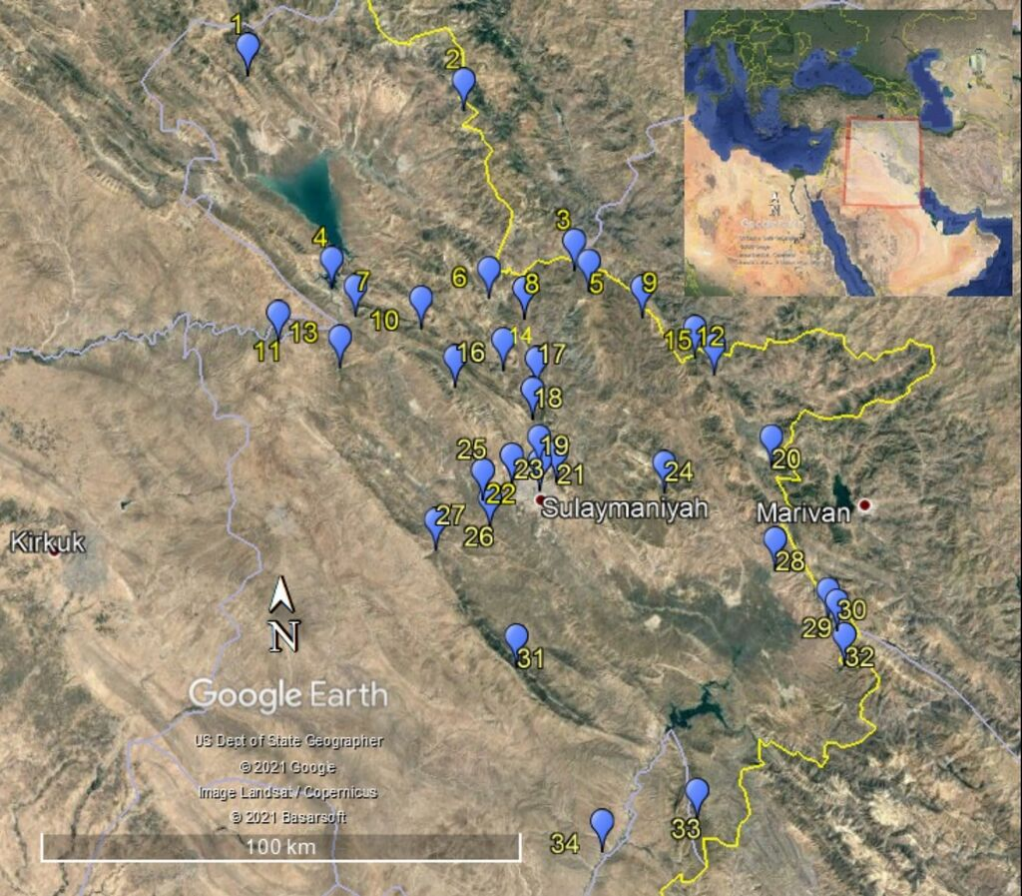
Map of Sulaymaniyah Province, the sampling localities marked with blue dots.

All butterflies were collected by F. Khudhur between April 2016 and April 2021. The specimens were collected from 34 different sites throughout the Sulaymaniyah Province (Table 1, Fig. 1). Sweeping nets were used for collecting the specimens. Specimens were euthanised with ethyl acetate vapour and were then put into a paper envelope and brought to the laboratory pinned, mounted, labelled and deposited in the Entomology Laboratory, Department of Biology, College of Science, University of Sulaimani, Sulaymaniyah Province, Kurdistan Region, Iraq. The species were identified according to Wiltshire (1957), Higgins (1958), Baytaş (2007), Eckweiler (2011), Tshikolovets et al. (2014), Tikhonov et al. (2020).

## Data resources

A total of 103 species of butterfly, belonging to five families of the superfamily Papilionoidea, order Lepidoptera, were identified. Of which eight species, previously unrecorded, were added as new species to the Iraqi butterfly fauna; these eight species are marked with an asterisk in the Checklist section. Five species were recorded within Papilionidae, 19 species within Hesperiidae including two new records, 18 species within Pieridae including three new records, 25 species within Lycaenidae with one new record and 36 species within Nymphalidae including two new records. The species of the butterflies were listed according to Wiemers and Verovnik (2016).

## Checklists

### Checklist of Butterflies of Sulaymaniyah Province, Kurdista Region, Iraq

#### Carcharodus alceae

(Esper, [1780])

47E5BD06-C0D7-5380-93D4-01CCFD8684A9

##### Materials

- **Type status:** Other material. **Location:** county: Pishdar; locality: Shênê Village; verbatimCoordinates: 36°17'00"N, 45°16'01"E
- **Type status:** Other material. **Location:** county: Dukan; locality: Upper Dukan; verbatimCoordinates: 35°56'59"N, 44°57'38"E
- **Type status:** Other material. **Location:** county: Chamchamal; locality: Goptapa Village; verbatimCoordinates: 35°51'00"N, 44°50'07"E
- **Type status:** Other material. **Location:** county: Dukan; locality: Chami Razan Valley; verbatimCoordinates: 35°48'03"N, 44°58'38"E
- **Type status:** Other material. **Location:** county: Sulyamaniyah; locality: Qlyasan; verbatimCoordinates: 35°34'41"N, 45°22'01"E
- **Type status:** Other material. **Location:** county: Bakrajo; locality: Hazarmerd; verbatimCoordinates: 35°29'56"N, 45°18'54"E
- **Type status:** Other material. **Location:** county: Bazyan; locality: Dêlêzha; verbatimCoordinates: 35°27'36"N, 45°11'26"E
- **Type status:** Other material. **Location:** county: Halabja; locality: Byara; verbatimCoordinates: 35°13'47"N, 46°07'13"E
- **Type status:** Other material. **Location:** county: Kalar; locality: Awa Khwery; verbatimCoordinates: 34°53'30"N, 45°33'29"E

#### Carcharodus orientalis

Reverdin, 1913

CB23C105-8CA9-5362-9FF4-D64743141542

##### Materials

- **Type status:** Other material. **Location:** county: Chuarta; locality: Upper Dêrê Village; verbatimCoordinates: 35°56'08"N, 44°57'38"E
- **Type status:** Other material. **Location:** county: Bakrajo; locality: Hazarmerd; verbatimCoordinates: 35°29'56"N, 45°18'54"E

#### * Carcharodus stauderi

Reverdin, 1913

C346BA24-6621-51DE-96F8-39371D13F584

##### Materials

- **Type status:** Other material. **Location:** county: Chwarta; municipality: Basnê; locality: Upper Dêrê; verbatimCoordinates: 35°56'08"N, 44°57'38"E; **Identification:** identifiedBy: Farhad A. Khudhur; identificationReferences: Tshikolovets et.al. 2014; **Event:** eventDate: 21-Jun-20; **Record Level:** basisOfRecord: PreservedSpecimen
- **Type status:** Other material. **Location:** county: Dukan; locality: Zêwê (Piramagroon Mount.); verbatimCoordinates: 35°45'41"N, 45°14'17"E; **Identification:** identifiedBy: Farhad A. Khudhur; identificationReferences: Tshikolovets etal. 2014; **Event:** eventDate: 10-Aug-20; **Record Level:** basisOfRecord: PreservedSpecimen

##### Notes

First record for Iraq

#### Eogenes alcides

(Herrich-Schäffer, [1852])

2D313E12-204D-58A1-9BAC-312E4545FA4A

##### Materials

- **Type status:** Other material. **Location:** county: Dukan; locality: Qamchukha Village; verbatimCoordinates: 35°53'51"N, 45°00'51"E
- **Type status:** Other material. **Location:** county: Mawat; locality: Mawat; verbatimCoordinates: 35°53'10"N, 45°23'59"E
- **Type status:** Other material. **Location:** county: Dukan; locality: Sargalw (Bargalw); verbatimCoordinates: 35°52'44"N, 45°09'49"E
- **Type status:** Other material. **Location:** county: Sulyamaniyah; locality: Hawary Shar Park; verbatimCoordinates: 35°36'41"N, 45°25'48"E
- **Type status:** Other material. **Location:** county: Sulyamaniyah; locality: Qlyasan; verbatimCoordinates: 35°34'41"N, 45°22'01"E

#### Erynnis marloyi

(Boisduval, [1834])

721F97B5-6EF6-5A92-ABCA-27C68D40B96E

##### Materials

- **Type status:** Other material. **Location:** county: Mawat; locality: Mawat; verbatimCoordinates: 35°53'10"N, 45°23'59"E
- **Type status:** Other material. **Location:** county: Dukan; locality: Zêwê (Piramagroon Mount.); verbatimCoordinates: 35°45'41"N, 45°14'17"E

#### Erynnis tages

(Linnaeus, 1758)

E50C6867-41F1-52B8-97B5-C68CEB82B944

##### Materials

- **Type status:** Other material. **Location:** county: Chuarta; locality: Shanakhsê Village; verbatimCoordinates: 35°58'37"N, 45°31'11"E
- **Type status:** Other material. **Location:** county: Mawat; locality: Galala Village; verbatimCoordinates: 35°53'58"N, 45°19'51"E
- **Type status:** Other material. **Location:** county: Chuarta; locality: Little Barê Village; verbatimCoordinates: 35°53'02"N, 45°40'07"E

#### Gegenes nostrodamus

(Fabricius, 1793)

C3742920-EC1C-5681-83A1-B2F7AC4D6307

##### Materials

- **Type status:** Other material. **Location:** county: Dukan; locality: Qamchukha Village; verbatimCoordinates: 35°53'51"N, 45°00'51"E
- **Type status:** Other material. **Location:** county: Sulyamaniyah; locality: Hawary Shar Park; verbatimCoordinates: 35°36'41"N, 45°25'48"E
- **Type status:** Other material. **Location:** county: Qareh Dagh; locality: Qareh Dagh Mount.; verbatimCoordinates: 35°14'27"N, 45°22'12"E

#### Gegenes pumilio

(Hoffmannsegg, 1804)

980DA679-1E1B-54FE-AEB1-955E9340269D

##### Materials

- **Type status:** Other material. **Location:** county: Qareh Dagh; locality: Qareh Dagh Mount.; verbatimCoordinates: 35°14'27"N, 45°22'12"E
- **Type status:** Other material. **Location:** county: Halabja; locality: Sargat Village; verbatimCoordinates: 35°17'34"N, 46°06'18"E

#### Muschampia tessellum

(Hb., 1803)

CF4DFB30-CC29-5F02-B5A2-CDD0B2AF055A

##### Materials

- **Type status:** Other material. **Location:** county: Bakrajo; locality: Hazarmerd; verbatimCoordinates: 35°29'56"N, 45°18'54"E

#### Muschampia nomas

(Lederer, 1855)

0F4F4752-AA89-5478-BC8A-4B2967C2FE35

##### Materials

- **Type status:** Other material. **Location:** county: Bakrajo; locality: Hazarmerd; verbatimCoordinates: 35°29'56"N, 45°18'54"E

#### Ochlodes sylvanus

(Esper, 1777)

9E6F9750-53A1-5D73-B484-75591F23ED05

##### Materials

- **Type status:** Other material. **Location:** county: Chuarta; locality: Upper Dêrê Village; verbatimCoordinates: 35°56'08"N, 44°57'38"E

#### Spialia osthelderi

(Pfeiffer, 1932)

E02D5E0F-AD44-51E8-93AF-B87575CD9A0A

##### Materials

- **Type status:** Other material. **Location:** county: Chuarta; locality: Upper Dêrê Village; verbatimCoordinates: 35°56'08"N, 44°57'38"E

#### Spialia doris

(Walker, 1870)

BC816D21-13B0-5A54-96E9-03494F635B44

##### Materials

- **Type status:** Other material. **Location:** county: Pishdar; locality: Shênê Village; verbatimCoordinates: 36°17'00"N, 45°16'01"E
- **Type status:** Other material. **Location:** county: Mawat; locality: Mawat; verbatimCoordinates: 35°53'10"N, 45°23'59"E

#### Spialia geron

(Watson, 1893)

DD1AA476-3466-5D23-8E1C-1670835741B1

##### Materials

- **Type status:** Other material. **Location:** county: Sulyamaniyah; locality: Azady Park; verbatimCoordinates: 35°34'02"N, 45°25'51"E

#### Spialia orbifer

(Hübner, [1823])

F62984C9-9DE4-5349-B0C1-109301E60BBA

##### Materials

- **Type status:** Other material. **Location:** county: Dukan; locality: Sargalw (Bargalw); verbatimCoordinates: 35°52'44"N, 45°09'49"E

#### Thymelicus acteon

(Rottemburg, 1775)

C8B2A13B-05DD-5667-994E-D63F726806D7

##### Materials

- **Type status:** Other material. **Location:** county: Dukan; locality: Sargalw (Bargalw); verbatimCoordinates: 35°52'44"N, 45°09'49"E
- **Type status:** Other material. **Location:** county: Qareh Dagh; locality: Qareh Dagh Mount.; verbatimCoordinates: 35°14'27"N, 45°22'12"E

#### Thymelicus hyrax

(Lederer, 1861)

F50B8E52-B0AC-5882-85DF-12A28A4CCA0A

##### Materials

- **Type status:** Other material. **Occurrence:** recordedBy: F. A. Khudhur; sex: 2 males; **Location:** county: Bakrajo; municipality: Bakrajo; locality: Hazarmerd; verbatimCoordinates: 35°29'56"N, 45°18'54"E; **Identification:** identifiedBy: Farhad A. Khudhur; identificationReferences: Tshikolovets et al. 2014 & Baytaş 2007; **Event:** eventDate: 14-May-20; **Record Level:** basisOfRecord: PreservedSpecimen

##### Notes

First record for Iraq

#### Thymelicus lineola

(Ochsenheimer, 1808)

AB50C740-1013-5567-86BD-3124B95D6F93

##### Materials

- **Type status:** Other material. **Location:** county: Chuarta; locality: Shanakhsê Village; verbatimCoordinates: 35°58'37"N, 45°31'11"E
- **Type status:** Other material. **Location:** county: Dukan; locality: Sargalw (Bargalw); verbatimCoordinates: 35°52'44"N, 45°09'49"E
- **Type status:** Other material. **Location:** county: Qareh Dagh; locality: Qareh Dagh Mount.; verbatimCoordinates: 35°14'27"N, 45°22'12"E

#### Thymelicus sylvestris

(Poda, 1761)

9A467315-09EF-5FB8-9A94-0B1F2B91E7E4

##### Materials

- **Type status:** Other material. **Location:** county: Sulyamaniyah; locality: Hawary Shar Park; verbatimCoordinates: 35°36'41"N, 45°25'48"E
- **Type status:** Other material. **Location:** county: Sulyamaniyah; locality: Goyzha; verbatimCoordinates: 35°34'57"N, 45°28'09"E
- **Type status:** Other material. **Location:** county: Bakrajo; locality: Hazarmerd; verbatimCoordinates: 35°29'56"N, 45°18'54"E
- **Type status:** Other material. **Location:** county: Halabja; locality: Zalm Village; verbatimCoordinates: 35°18'53"N, 46°05'07"E

#### Archon apollinaris

(Staudinger, [1892])

08518401-1FDC-5CD5-9657-D8F9779F5016

##### Materials

- **Type status:** Other material. **Location:** county: Dukan; locality: Upper Dukan; verbatimCoordinates: 35°56'59"N, 44°57'38"E
- **Type status:** Other material. **Location:** county: Chamchamal; locality: Goptapa Village; verbatimCoordinates: 35°51'00"N, 44°50'07"E
- **Type status:** Other material. **Location:** county: Dukan; locality: Chami Razan Valley; verbatimCoordinates: 35°48'03"N, 44°58'38"E
- **Type status:** Other material. **Location:** county: Bakrajo; locality: Hazarmerd; verbatimCoordinates: 35°29'56"N, 45°18'54"E

#### Zerynthia deyrollei

(Oberthür, 1869)

A1BC242D-8C3E-58AD-A76B-1C6BB1CA8872

##### Materials

- **Type status:** Other material. **Location:** county: Dukan; locality: Upper Dukan; verbatimCoordinates: 35°56'59"N, 44°57'38"E
- **Type status:** Other material. **Location:** county: Chamchamal; locality: Goptapa Village; verbatimCoordinates: 35°51'00"N, 44°50'07"E
- **Type status:** Other material. **Location:** county: Dukan; locality: Chami Razan Valley; verbatimCoordinates: 35°48'03"N, 44°58'38"E
- **Type status:** Other material. **Location:** county: Sulyamaniyah; locality: Qlyasan; verbatimCoordinates: 35°34'41"N, 45°22'01"E
- **Type status:** Other material. **Location:** county: Bakrajo; locality: Hazarmerd; verbatimCoordinates: 35°29'56"N, 45°18'54"E

#### Iphiclides podalirius

(Linnaeus, 1758)

9E7FA5B6-8FF2-52F2-8F73-355160292BCF

##### Materials

- **Type status:** Other material. **Location:** county: Ranya; locality: Sarkapkan; verbatimCoordinates: 36°21'04"N, 44°46'24"E
- **Type status:** Other material. **Location:** county: Pishdar; locality: Shênê Village; verbatimCoordinates: 36°17'00"N, 45°16'01"E
- **Type status:** Other material. **Location:** county: Chuarta; locality: Shanakhsê Village; verbatimCoordinates: 35°58'37"N, 45°31'11"E
- **Type status:** Other material. **Location:** county: Chuarta; locality: Upper Dêrê Village; verbatimCoordinates: 35°56'08"N, 44°57'38"E
- **Type status:** Other material. **Location:** county: Mawat; locality: Galala Village; verbatimCoordinates: 35°53'58"N, 45°19'51"E
- **Type status:** Other material. **Location:** county: Penjwen; locality: Sya Gwez Village; verbatimCoordinates: 35°48'37"N, 45°47'33"E
- **Type status:** Other material. **Location:** county: Dukan; locality: Zêwê (Piramagroon Mount.); verbatimCoordinates: 35°45'41"N, 45°14'17"E
- **Type status:** Other material. **Location:** county: Qareh Dagh; locality: Qareh Dagh Mount.; verbatimCoordinates: 35°14'27"N, 45°22'12"E
- **Type status:** Other material. **Location:** county: Halabja; locality: Byara; verbatimCoordinates: 35°13'47"N, 46°07'13"E

#### Papilio demoleus

Linnaeus, 1758

4CB87121-22CD-5697-9FEE-3CBD4EB2723D

##### Materials

- **Type status:** Other material. **Location:** county: Sulyamaniyah; locality: Hawary Shar Park; verbatimCoordinates: 35°36'41"N, 45°25'48"E
- **Type status:** Other material. **Location:** county: Sulyamaniyah; locality: Qlyasan; verbatimCoordinates: 35°34'41"N, 45°22'01"E
- **Type status:** Other material. **Location:** county: Sulyamaniyah; locality: Azady Park; verbatimCoordinates: 35°34'02"N, 45°25'51"E
- **Type status:** Other material. **Location:** county: Kalar; locality: Awa Khwery; verbatimCoordinates: 34°53'30"N, 45°33'29"E

#### Papilio machaon

Linnaeus, 1758

8CECF23E-2EEF-527C-892A-8DB984EC6C94

##### Materials

- **Type status:** Other material. **Location:** county: Mawat; locality: Galala Village; verbatimCoordinates: 35°53'58"N, 45°19'51"E
- **Type status:** Other material. **Location:** county: Dukan; locality: Zêwê (Piramagroon Mount.); verbatimCoordinates: 35°45'41"N, 45°14'17"E
- **Type status:** Other material. **Location:** county: Sulyamaniyah; locality: Azady Park; verbatimCoordinates: 35°34'02"N, 45°25'51"E
- **Type status:** Other material. **Location:** county: Qareh Dagh; locality: Qareh Dagh Mount.; verbatimCoordinates: 35°14'27"N, 45°22'12"E

#### Anthocharis gruneri

(Herrich-Schaffer, [1851])

0A4FBE97-C41A-521A-972B-2FC9AA6B7A19

##### Materials

- **Type status:** Other material. **Location:** county: Ranya; locality: Sarkapkan; verbatimCoordinates: 36°21'04"N, 44°46'24"E

#### Anthocharis cardamines

(Linnaeus, 1758)

AA45CC9D-29FF-5838-8A85-C202FF1F4D73

##### Materials

- **Type status:** Other material. **Location:** county: Bakrajo; locality: Hazarmerd; verbatimCoordinates: 35°29'56"N, 45°18'54"E

#### Aporia crataegi

(Linnaeus, 1758)

1587F301-609C-5138-985D-4371CD78E4B6

##### Materials

- **Type status:** Other material. **Location:** county: Chamchamal; locality: Goptapa Village; verbatimCoordinates: 35°51'00"N, 44°50'07"E
- **Type status:** Other material. **Location:** county: Bakrajo; locality: Hazarmerd; verbatimCoordinates: 35°29'56"N, 45°18'54"E
- **Type status:** Other material. **Location:** county: Bazyan; locality: Dêlêzha; verbatimCoordinates: 35°27'36"N, 45°11'26"E

#### Belenois aurota

(Fabricius, 1793)

B59C59C9-282B-5A7A-9654-504290F3355B

##### Materials

- **Type status:** Other material. **Location:** county: Dukan; locality: Qamchukha Village; verbatimCoordinates: 35°53'51"N, 45°00'51"E
- **Type status:** Other material. **Location:** county: Sulyamaniyah; locality: Qlyasan; verbatimCoordinates: 35°34'41"N, 45°22'01"E
- **Type status:** Other material. **Location:** county: Bakrajo; locality: Kany Pan; verbatimCoordinates: 35°33'03"N, 45°18'00"E

#### Colias croceus

(Geoffroy, 1785)

005550A4-19F0-5FD5-842A-B651B275CA20

##### Materials

- **Type status:** Other material. **Location:** county: Pishdar; locality: Shênê Village; verbatimCoordinates: 36°17'00"N, 45°16'01"E
- **Type status:** Other material. **Location:** county: Dukan; locality: Qamchukha Village; verbatimCoordinates: 35°53'51"N, 45°00'51"E
- **Type status:** Other material. **Location:** county: Mawat; locality: Mawat; verbatimCoordinates: 35°53'10"N, 45°23'59"E
- **Type status:** Other material. **Location:** county: Chuarta; locality: Little Barê Village; verbatimCoordinates: 35°53'02"N, 45°40'07"E
- **Type status:** Other material. **Location:** county: Mawat; locality: Mokaba; verbatimCoordinates: 35°45'26"N, 45°25'41"E
- **Type status:** Other material. **Location:** county: Sulyamaniyah; locality: Hawary Shar Park; verbatimCoordinates: 35°36'41"N, 45°25'48"E
- **Type status:** Other material. **Location:** county: Sulyamaniyah; locality: Azady Park; verbatimCoordinates: 35°34'02"N, 45°25'51"E
- **Type status:** Other material. **Location:** county: Qareh Dagh; locality: Qareh Dagh Mount.; verbatimCoordinates: 35°14'27"N, 45°22'12"E

#### * Colias erate

(Esper, 1805)

8CA274E5-AEB3-5FC8-84E7-D9215F75699C

##### Materials

- **Type status:** Other material. **Occurrence:** recordedBy: F. A. Khudhur; sex: 1 male, 2 females; **Location:** county: Qareh Dagh; locality: Qareh Dagh Mount.; verbatimCoordinates: 35°14'27"N, 45°22'12"E; **Identification:** identifiedBy: Farhad A. Khudhur; identificationReferences: Tshikolovets etal. 2014; **Event:** eventDate: 12-Sep-20; **Record Level:** basisOfRecord: PreservedSpecimen

##### Notes

First record for Iraq

#### Colias aurorina

Herrich-Schäffer, 1850

FFD6851D-DEFB-55B2-9E87-4DF57BC44A5F

##### Materials

- **Type status:** Other material. **Location:** county: Mawat; locality: Galala Village; verbatimCoordinates: 35°53'58"N, 45°19'51"E
- **Type status:** Other material. **Location:** county: Sulyamaniyah; locality: Hawary Shar Park; verbatimCoordinates: 35°36'41"N, 45°25'48"E

#### Colotis fausta

(Olivier, [1804])

38F8DA10-E271-56A7-8D90-0E9D7D0C256D

##### Materials

- **Type status:** Other material. **Location:** county: Dukan; locality: Qamchukha Village; verbatimCoordinates: 35°53'51"N, 45°00'51"E
- **Type status:** Other material. **Location:** county: Sulyamaniyah; locality: Qlyasan; verbatimCoordinates: 35°34'41"N, 45°22'01"E
- **Type status:** Other material. **Location:** county: Bakrajo; locality: Kany Pan; verbatimCoordinates: 35°33'03"N, 45°18'00"E
- **Type status:** Other material. **Location:** county: Darbandikhan; locality: Sartak; verbatimCoordinates: 34°56'45"N, 45°46'32"E
- **Type status:** Other material. **Location:** county: Kalar; locality: Awa Khwery; verbatimCoordinates: 34°53'30"N, 45°33'29"E

#### Euchloe belemia

(Esper, 1800)

A17472A5-6CBE-533A-A89E-F46024C37844

##### Materials

- **Type status:** Other material. **Location:** county: Penjwen; locality: Penjwen; verbatimCoordinates: 35°36'09"N, 45°57'48"E

#### * Gonepteryx rhamni

(Linnaeus, 1758)

D6DA5F04-D749-5DDA-917F-9626F54DAFEC

##### Materials

- **Type status:** Other material. **Occurrence:** recordedBy: F. A. Khudhur; sex: 2 males; **Location:** county: Dukan; locality: Zêwê (Piramagroon Mount.); verbatimCoordinates: 35°45'41"N, 45°14'17"E; **Identification:** identifiedBy: Farhad A. Khudhur; identificationReferences: Tshikolovets etal. 2014; **Event:** eventDate: 4-May-21; **Record Level:** basisOfRecord: PreservedSpecimen

##### Notes

First record for Iraq

#### Gonepteryx farinosa

(Zeller, 1847)

ABCEF433-40CF-5887-869B-D19D147E6FC9

##### Materials

- **Type status:** Other material. **Location:** county: Chuarta; locality: Upper Dêrê Village; verbatimCoordinates: 35°56'08"N, 44°57'38"E
- **Type status:** Other material. **Location:** county: Mawat; locality: Galala Village; verbatimCoordinates: 35°53'58"N, 45°19'51"E
- **Type status:** Other material. **Location:** county: Qareh Dagh; locality: Qareh Dagh Mount.; verbatimCoordinates: 35°14'27"N, 45°22'12"E

#### Pieris ergane

(Geyer, [1828])

81DCDB13-81C9-59F7-AE24-E02FC9EEF4D8

##### Materials

- **Type status:** Other material. **Location:** county: Halabja; locality: Sargat Village; verbatimCoordinates: 35°17'34"N, 46°06'18"E

#### * Pieris krueperi

Staudinger, 1860

D9D64BCB-8AEB-5EB5-9F65-091424FB3838

##### Materials

- **Type status:** Other material. **Occurrence:** recordedBy: F. A. Khudhur; sex: 1 male; **Location:** county: Said Sadiq; locality: Nawê Village; verbatimCoordinates: 35°24'42"N, 45°57'59"E; **Identification:** identifiedBy: Farhad A. Khudhur; identificationReferences: Tshikolovets etal. 2014; **Event:** eventDate: 27-Oct-17; **Record Level:** basisOfRecord: PreservedSpecimen

##### Notes

First record for Iraq

#### Pieris napi

(Linnaeus, 1758)

501E98B6-55BB-5735-9C4E-6400BADECD71

##### Materials

- **Type status:** Other material. **Location:** county: Sulyamaniyah; locality: Qlyasan; verbatimCoordinates: 35°34'41"N, 45°22'01"E
- **Type status:** Other material. **Location:** county: Halabja; locality: Byara; verbatimCoordinates: 35°13'47"N, 46°07'13"E

#### Pieris rapae

(Linnaeus, 1758)

652E4B83-81F8-555E-BB7B-42D9B82F4A2D

##### Materials

- **Type status:** Other material. **Location:** county: Ranya; locality: Sarkapkan; verbatimCoordinates: 36°21'04"N, 44°46'24"E
- **Type status:** Other material. **Location:** county: Pishdar; locality: Shênê Village; verbatimCoordinates: 36°17'00"N, 45°16'01"E
- **Type status:** Other material. **Location:** county: Dukan; locality: Upper Dukan; verbatimCoordinates: 35°56'59"N, 44°57'38"E
- **Type status:** Other material. **Location:** county: Mawat; locality: Mawat; verbatimCoordinates: 35°53'10"N, 45°23'59"E
- **Type status:** Other material. **Location:** county: Chamchamal; locality: Goptapa Village; verbatimCoordinates: 35°51'00"N, 44°50'07"E
- **Type status:** Other material. **Location:** county: Dukan; locality: Chami Razan Valley; verbatimCoordinates: 35°48'03"N, 44°58'38"E
- **Type status:** Other material. **Location:** county: Mawat; locality: Mokaba; verbatimCoordinates: 35°45'26"N, 45°25'41"E
- **Type status:** Other material. **Location:** county: Mawat; locality: Qaiwan Village; verbatimCoordinates: 35°42'01"N, 45°25'03"E
- **Type status:** Other material. **Location:** county: Penjwen; locality: Penjwen; verbatimCoordinates: 35°36'09"N, 45°57'48"E
- **Type status:** Other material. **Location:** county: Sulyamaniyah; locality: Qlyasan; verbatimCoordinates: 35°34'41"N, 45°22'01"E
- **Type status:** Other material. **Location:** county: Bakrajo; locality: Kany Pan; verbatimCoordinates: 35°33'03"N, 45°18'00"E
- **Type status:** Other material. **Location:** county: Bazyan; locality: Dêlêzha; verbatimCoordinates: 35°27'36"N, 45°11'26"E
- **Type status:** Other material. **Location:** county: Halabja; locality: Byara; verbatimCoordinates: 35°13'47"N, 46°07'13"E

#### Pontia chloridice

(Hübner, [1808-1813])

59902F06-7A2D-507F-B056-AF9855F85BCB

##### Materials

- **Type status:** Other material. **Location:** county: Barzinja; locality: Basak Village; verbatimCoordinates: 35°33'30"N, 45°42'57"E

#### Pontia glauconome

Klug, 1829

6E7F946C-A1E1-5B44-83D0-FECBE97F577A

##### Materials

- **Type status:** Other material. **Location:** county: Dukan; locality: Sargalw (Bargalw); verbatimCoordinates: 35°52'44"N, 45°09'49"E
- **Type status:** Other material. **Location:** county: Bakrajo; locality: Hazarmerd; verbatimCoordinates: 35°29'56"N, 45°18'54"E

#### Pontia daplidice

(Linnaeus, 1758)

BBE7AEBC-D119-570B-A4FA-112A6EA15CB1

##### Materials

- **Type status:** Other material. **Location:** county: Ranya; locality: Sarkapkan; verbatimCoordinates: 36°21'04"N, 44°46'24"E
- **Type status:** Other material. **Location:** county: Pishdar; locality: Shênê Village; verbatimCoordinates: 36°17'00"N, 45°16'01"E
- **Type status:** Other material. **Location:** county: Mawat; locality: Galala Village; verbatimCoordinates: 35°53'58"N, 45°19'51"E
- **Type status:** Other material. **Location:** county: Mawat; locality: Mokaba; verbatimCoordinates: 35°45'26"N, 45°25'41"E
- **Type status:** Other material. **Location:** county: Bakrajo; locality: Kany Pan; verbatimCoordinates: 35°33'03"N, 45°18'00"E
- **Type status:** Other material. **Location:** county: Bakrajo; locality: Hazarmerd; verbatimCoordinates: 35°29'56"N, 45°18'54"E
- **Type status:** Other material. **Location:** county: Qareh Dagh; locality: Qareh Dagh Mount.; verbatimCoordinates: 35°14'27"N, 45°22'12"E

#### Aricia agestis

(Denis & Schiffermüller, 1775)

9348651C-D295-5D87-B5D3-87EF8948C866

##### Materials

- **Type status:** Other material. **Location:** county: Mawat; locality: Mawat; verbatimCoordinates: 35°53'10"N, 45°23'59"E
- **Type status:** Other material. **Location:** county: Sulyamaniyah; locality: Qlyasan; verbatimCoordinates: 35°34'41"N, 45°22'01"E
- **Type status:** Other material. **Location:** county: Barzinja; locality: Basak Village; verbatimCoordinates: 35°33'30"N, 45°42'57"E
- **Type status:** Other material. **Location:** county: Bakrajo; locality: Kany Pan; verbatimCoordinates: 35°33'03"N, 45°18'00"E
- **Type status:** Other material. **Location:** county: Bakrajo; locality: Hazarmerd; verbatimCoordinates: 35°29'56"N, 45°18'54"E
- **Type status:** Other material. **Location:** county: Bazyan; locality: Dêlêzha; verbatimCoordinates: 35°27'36"N, 45°11'26"E
- **Type status:** Other material. **Location:** county: Said Sadiq; locality: Nawê Village; verbatimCoordinates: 35°24'42"N, 45°57'59"E
- **Type status:** Other material. **Location:** county: Halabja; locality: Zalm Village; verbatimCoordinates: 35°18'53"N, 46°05'07"E
- **Type status:** Other material. **Location:** county: Halabja; locality: Byara; verbatimCoordinates: 35°13'47"N, 46°07'13"E

#### Celastrina (44) argiolus

(Linnaeus, 1758)

BC12FB61-8309-50C2-A7A1-EF4A006E71DB

##### Materials

- **Type status:** Other material. **Location:** county: Dukan; locality: Zêwê (Piramagroon Mount.); verbatimCoordinates: 35°45'41"N, 45°14'17"E

#### Cigaritis maxima

Staudinger, 1901

DA743377-A90C-5A4C-8D3E-A88794DEAF9D

##### Materials

- **Type status:** Other material. **Location:** county: Mawat; locality: Mawat; verbatimCoordinates: 35°53'10"N, 45°23'59"E

#### Cigaritis epargyros

(Eversmann, 1854)

4314CC83-00E0-5672-9455-146F46EBDF82

##### Materials

- **Type status:** Other material. **Location:** county: Mawat; locality: Mawat; verbatimCoordinates: 35°53'10"N, 45°23'59"E
- **Type status:** Other material. **Location:** county: Mawat; locality: Balkha Village; verbatimCoordinates: 35°47'09"N, 45°22'47"E

#### Favonius quercus

(Linnaeus, 1758)

290E6791-658D-5FDF-B4F7-32AA42D064A3

##### Materials

- **Type status:** Other material. **Location:** county: Dukan; locality: Zêwê (Piramagroon Mount.); verbatimCoordinates: 35°45'41"N, 45°14'17"E

#### Glaucopsyche laetifica safidensis

(Blom, 1979)

0EAB179E-3C96-56FD-9ED5-F49CBE2DD4A3

##### Materials

- **Type status:** Other material. **Location:** county: Mawat; locality: Mokaba; verbatimCoordinates: 35°45'26"N, 45°25'41"E
- **Type status:** Other material. **Location:** county: Bakrajo; locality: Hazarmerd; verbatimCoordinates: 35°29'56"N, 45°18'54"E

#### Lampides boeticus

(Linnaeus, 1767)

2B9088EE-D9AB-5011-8157-98B0373534AC

##### Materials

- **Type status:** Other material. **Location:** county: Mawat; locality: Mawat; verbatimCoordinates: 35°53'10"N, 45°23'59"E
- **Type status:** Other material. **Location:** county: Penjwen; locality: Gollê Resort; verbatimCoordinates: 35°46'31"N, 45°49'54"E
- **Type status:** Other material. **Location:** county: Sulyamaniyah; locality: Goyzha; verbatimCoordinates: 35°34'57"N, 45°28'09"E
- **Type status:** Other material. **Location:** county: Bakrajo; locality: Kany Pan; verbatimCoordinates: 35°33'03"N, 45°18'00"E
- **Type status:** Other material. **Location:** county: Bazyan; locality: Dêlêzha; verbatimCoordinates: 35°27'36"N, 45°11'26"E
- **Type status:** Other material. **Location:** county: Kalar; locality: Awa Khwery; verbatimCoordinates: 34°53'30"N, 45°33'29"E

#### Chilades trochylus

(Freyer, 1845)

17B59791-23B4-5F59-96A1-288EE0DACAFB

##### Materials

- **Type status:** Other material. **Location:** county: Sulyamaniyah; locality: Hawary Shar Park; verbatimCoordinates: 35°36'41"N, 45°25'48"E
- **Type status:** Other material. **Location:** county: Sulyamaniyah; locality: Azady Park; verbatimCoordinates: 35°34'02"N, 45°25'51"E
- **Type status:** Other material. **Location:** county: Bakrajo; locality: Hazarmerd; verbatimCoordinates: 35°29'56"N, 45°18'54"E

#### Lachides galba

(Lederer, 1855)

CED16726-EA1D-5F03-BF05-86D3DB990573

##### Materials

- **Type status:** Other material. **Location:** county: Sulyamaniyah; locality: Hawary Shar Park; verbatimCoordinates: 35°36'41"N, 45°25'48"E
- **Type status:** Other material. **Location:** county: Sulyamaniyah; locality: Qlyasan; verbatimCoordinates: 35°34'41"N, 45°22'01"E
- **Type status:** Other material. **Location:** county: Sulyamaniyah; locality: Azady Park; verbatimCoordinates: 35°34'02"N, 45°25'51"E
- **Type status:** Other material. **Location:** county: Bakrajo; locality: Kany Pan; verbatimCoordinates: 35°33'03"N, 45°18'00"E

#### Lycaena asabinus

(Herrich-Schaffer, [1851])

1CBDED23-CFAC-505D-8D33-55C42F7B13FC

##### Materials

- **Type status:** Other material. **Location:** county: Pishdar; locality: Shênê Village; verbatimCoordinates: 36°17'00"N, 45°16'01"E
- **Type status:** Other material. **Location:** county: Dukan; locality: Zêwê (Piramagroon Mount.); verbatimCoordinates: 35°45'41"N, 45°14'17"E

#### Lycaena phlaeas

(Linnaeus, 1761)

C068E71C-7327-53FD-9030-0BAF3FDAE6D0

##### Materials

- **Type status:** Other material. **Location:** county: Pishdar; locality: Shênê Village; verbatimCoordinates: 36°17'00"N, 45°16'01"E
- **Type status:** Other material. **Location:** county: Mawat; locality: Mawat; verbatimCoordinates: 35°53'10"N, 45°23'59"E
- **Type status:** Other material. **Location:** county: Dukan; locality: Zêwê (Piramagroon Mount.); verbatimCoordinates: 35°45'41"N, 45°14'17"E
- **Type status:** Other material. **Location:** county: Sulyamaniyah; locality: Qlyasan; verbatimCoordinates: 35°34'41"N, 45°22'01"E

#### Lycaena thersamon

(Esper, 1784)

8B89871B-CC3E-5978-B598-0721B3B22C30

##### Materials

- **Type status:** Other material. **Location:** county: Chuarta; locality: Upper Dêrê Village; verbatimCoordinates: 35°56'08"N, 44°57'38"E
- **Type status:** Other material. **Location:** county: Sulyamaniyah; locality: Goyzha; verbatimCoordinates: 35°34'57"N, 45°28'09"E

#### Lycaena tityrus

(Poda, 1761)

201352C4-0BDC-5668-88BE-5C00AB616FC5

##### Materials

- **Type status:** Other material. **Location:** county: Chuarta; locality: Upper Dêrê Village; verbatimCoordinates: 35°56'08"N, 44°57'38"E
- **Type status:** Other material. **Location:** county: Penjwen; locality: Gollê Resort; verbatimCoordinates: 35°46'31"N, 45°49'54"E

#### Lycaena thetis

Klug, 1834

6FD55369-A778-572E-A173-C1AABA0BAD9F

##### Materials

- **Type status:** Other material. **Location:** county: Mawat; locality: Mawat; verbatimCoordinates: 35°53'10"N, 45°23'59"E

#### Plebejus zephyrinus

(Christoph, 1884)

AA1E221F-0CC2-55EA-BB93-8FD64D33B241

##### Materials

- **Type status:** Other material. **Location:** county: Chuarta; locality: Upper Dêrê Village; verbatimCoordinates: 35°56'08"N, 44°57'38"E
- **Type status:** Other material. **Location:** county: Sulyamaniyah; locality: Hawary Shar Park; verbatimCoordinates: 35°36'41"N, 45°25'48"E
- **Type status:** Other material. **Location:** county: Sulyamaniyah; locality: Goyzha; verbatimCoordinates: 35°34'57"N, 45°28'09"E

#### Polyommatus (Lysandra) bellargus

von Rottemburg, 1775

48698AF7-2915-59F6-BC34-DE237D84E18C

##### Materials

- **Type status:** Other material. **Location:** county: Chuarta; locality: Shanakhsê Village; verbatimCoordinates: 35°58'37"N, 45°31'11"E
- **Type status:** Other material. **Location:** county: Qareh Dagh; locality: Qareh Dagh Mount.; verbatimCoordinates: 35°14'27"N, 45°22'12"E

#### Polyommatus daphnis

(Denis & Schiffermuller, 1775)

3335AE52-2404-556B-A74B-508B04D5474F

##### Materials

- **Type status:** Other material. **Location:** county: Penjwen; locality: Sya Gwez Village; verbatimCoordinates: 35°48'37"N, 45°47'33"E
- **Type status:** Other material. **Location:** county: Dukan; locality: Zêwê (Piramagroon Mount.); verbatimCoordinates: 35°45'41"N, 45°14'17"E

#### Polyommatus icarus

(Rottemburg, 1775)

D33AB2A0-BD05-56F0-8CF3-06E220EBDCB2

##### Materials

- **Type status:** Other material. **Location:** county: Pishdar; locality: Shênê Village; verbatimCoordinates: 36°17'00"N, 45°16'01"E
- **Type status:** Other material. **Location:** county: Chuarta; locality: Upper Dêrê Village; verbatimCoordinates: 35°56'08"N, 44°57'38"E

#### * Polyommatus thersites

(Cantener, 1835)

67CCC776-F211-509D-A227-5FD01D4BD5F8

##### Materials

- **Type status:** Other material. **Occurrence:** recordedBy: F. A. Khudhur; sex: 1 male; **Location:** county: Barzinja; locality: Basak Village; verbatimCoordinates: 35°33'30"N, 45°42'57"E; **Identification:** identifiedBy: Farhad A. Khudhur; identificationReferences: Tshikolovets etal. 2014; **Event:** eventDate: 5-May-16; **Record Level:** basisOfRecord: PreservedSpecimen

##### Notes

First record for Iraq

#### Pseudophilotes vicrama

(Moore, 1865)

E44CEE20-A1F4-567F-BBDF-E9435B4B30CB

##### Materials

- **Type status:** Other material. **Location:** county: Pishdar; locality: Shênê Village; verbatimCoordinates: 36°17'00"N, 45°16'01"E

#### Satyrium acaciae

(Fabricius 1787).

D42B3D57-1B27-5CF1-BD84-0D944F7A35D3

##### Materials

- **Type status:** Other material. **Location:** county: Dukan; locality: Zêwê (Piramagroon Mount.); verbatimCoordinates: 35°45'41"N, 45°14'17"E

#### Satyrium marcidus

(Riley, 1921)

4AAC4DC0-6C1F-5A63-8632-AE63BE318C8D

##### Materials

- **Type status:** Other material. **Location:** county: Mawat; locality: Galala Village; verbatimCoordinates: 35°53'58"N, 45°19'51"E
- **Type status:** Other material. **Location:** county: Qareh Dagh; locality: Qareh Dagh Mount.; verbatimCoordinates: 35°14'27"N, 45°22'12"E

#### Satyrium abdominalis

(Gerhard, 1850)

8F581935-81D0-595F-801A-F132493814C5

##### Materials

- **Type status:** Other material. **Location:** county: Dukan; locality: Sargalw (Bargalw); verbatimCoordinates: 35°52'44"N, 45°09'49"E
- **Type status:** Other material. **Location:** county: Dukan; locality: Zêwê (Piramagroon Mount.); verbatimCoordinates: 35°45'41"N, 45°14'17"E

#### Tarucus balkanicus

(Freyer, [1844])

81AAF4DD-94AD-509E-A6E9-99E11C445BCC

##### Materials

- **Type status:** Other material. **Location:** county: Pishdar; locality: Shênê Village; verbatimCoordinates: 36°17'00"N, 45°16'01"E
- **Type status:** Other material. **Location:** county: Mawat; locality: Mawat; verbatimCoordinates: 35°53'10"N, 45°23'59"E
- **Type status:** Other material. **Location:** county: Dukan; locality: Sargalw (Bargalw); verbatimCoordinates: 35°52'44"N, 45°09'49"E
- **Type status:** Other material. **Location:** county: Bakrajo; locality: Hazarmerd; verbatimCoordinates: 35°29'56"N, 45°18'54"E
- **Type status:** Other material. **Location:** county: Bazyan; locality: Dêlêzha; verbatimCoordinates: 35°27'36"N, 45°11'26"E
- **Type status:** Other material. **Location:** county: Qareh Dagh; locality: Qareh Dagh Mount.; verbatimCoordinates: 35°14'27"N, 45°22'12"E

#### Zizeeria karsandra

(Moore, 1865)

9BB4A2A6-C5BF-5E85-901F-C65DBC83B4F9

##### Materials

- **Type status:** Other material. **Location:** county: Dukan; locality: Qamchukha Village; verbatimCoordinates: 35°53'51"N, 45°00'51"E
- **Type status:** Other material. **Location:** county: Sulyamaniyah; locality: Azady Park; verbatimCoordinates: 35°34'02"N, 45°25'51"E

#### Argynnis niobe

von Rottemburg, 1775

166A5327-4E20-5D07-A8C6-73617D6A5B95

##### Materials

- **Type status:** Other material. **Location:** county: Qareh Dagh; locality: Qareh Dagh Mount.; verbatimCoordinates: 35°14'27"N, 45°22'12"E

#### Argynnis pandora

([Schiffermüller], 1775)

04519FA6-BE5D-5A27-97B3-1C9F16A71F35

##### Materials

- **Type status:** Other material. **Location:** county: Ranya; locality: Sarkapkan; verbatimCoordinates: 36°21'04"N, 44°46'24"E
- **Type status:** Other material. **Location:** county: Pishdar; locality: Shênê Village; verbatimCoordinates: 36°17'00"N, 45°16'01"E
- **Type status:** Other material. **Location:** county: Chuarta; locality: Upper Dêrê Village; verbatimCoordinates: 35°56'08"N, 44°57'38"E
- **Type status:** Other material. **Location:** county: Penjwen; locality: Sya Gwez Village; verbatimCoordinates: 35°48'37"N, 45°47'33"E
- **Type status:** Other material. **Location:** county: Dukan; locality: Zêwê (Piramagroon Mount.); verbatimCoordinates: 35°45'41"N, 45°14'17"E
- **Type status:** Other material. **Location:** county: Qareh Dagh; locality: Qareh Dagh Mount.; verbatimCoordinates: 35°14'27"N, 45°22'12"E
- **Type status:** Other material. **Location:** county: Halabja; locality: Byara; verbatimCoordinates: 35°13'47"N, 46°07'13"E

#### * Brenthis mofidii

Wyatt, 1969

ED90C0D3-E44D-59B6-8F06-749028AC093F

##### Materials

- **Type status:** Other material. **Occurrence:** recordedBy: F. A. Khudhur; sex: 1 male; **Location:** county: Chuarta; locality: Upper Dêrê Village; verbatimCoordinates: 35°56'08"N, 45°57'38"E; **Identification:** identifiedBy: Farhad A. Khudhur; identificationReferences: Tshikolovets etal. 2014; **Event:** eventDate: 21-Jun-20; **Record Level:** basisOfRecord: PreservedSpecimen

##### Notes

First record for Iraq.

#### Brintesia circe

(Fabricius, 1775)

E9ABA8D2-C1F8-5196-A48A-443C1A41EF3B

##### Materials

- **Type status:** Other material. **Location:** county: Chuarta; locality: Upper Dêrê Village; verbatimCoordinates: 35°56'08"N, 44°57'38"E
- **Type status:** Other material. **Location:** county: Mawat; locality: Galala Village; verbatimCoordinates: 35°53'58"N, 45°19'51"E
- **Type status:** Other material. **Location:** county: Sulyamaniyah; locality: Hawary Shar Park; verbatimCoordinates: 35°36'41"N, 45°25'48"E
- **Type status:** Other material. **Location:** county: Qareh Dagh; locality: Qareh Dagh Mount.; verbatimCoordinates: 35°14'27"N, 45°22'12"E

#### Chazara briseis

(Linnaeus, 1764)

2267F894-F92F-576E-B26D-0D16FE3BC8E5

##### Materials

- **Type status:** Other material. **Location:** county: Chuarta; locality: Upper Dêrê Village; verbatimCoordinates: 35°56'08"N, 44°57'38"E
- **Type status:** Other material. **Location:** county: Halabja; locality: Sargat Village; verbatimCoordinates: 35°17'34"N, 46°06'18"E

#### Coenonympha pamphilus

(Linnaeus, 1758)

5F5F3A7F-628C-5C49-A6D7-2FC79030A572

##### Materials

- **Type status:** Other material. **Location:** county: Chuarta; locality: Upper Dêrê Village; verbatimCoordinates: 35°56'08"N, 44°57'38"E
- **Type status:** Other material. **Location:** county: Mawat; locality: Galala Village; verbatimCoordinates: 35°53'58"N, 45°19'51"E
- **Type status:** Other material. **Location:** county: Mawat; locality: Mawat; verbatimCoordinates: 35°53'10"N, 45°23'59"E
- **Type status:** Other material. **Location:** county: Dukan; locality: Sargalw (Bargalw); verbatimCoordinates: 35°52'44"N, 45°09'49"E

#### Coenonympha saadi

Kollar, [1849]

8A30FD5C-C1BC-54C4-866F-3641CF155BFB

##### Materials

- **Type status:** Other material. **Location:** county: Chuarta; locality: Little Barê Village; verbatimCoordinates: 35°53'02"N, 45°40'07"E
- **Type status:** Other material. **Location:** county: Dukan; locality: Sargalw (Bargalw); verbatimCoordinates: 35°52'44"N, 45°09'49"E
- **Type status:** Other material. **Location:** county: Dukan; locality: Zêwê (Piramagroon Mount.); verbatimCoordinates: 35°45'41"N, 45°14'17"E
- **Type status:** Other material. **Location:** county: Bakrajo; locality: Hazarmerd; verbatimCoordinates: 35°29'56"N, 45°18'54"E
- **Type status:** Other material. **Location:** county: Qareh Dagh; locality: Qareh Dagh Mount.; verbatimCoordinates: 35°14'27"N, 45°22'12"E

#### Danaus chrysippus

(Linnaeus, 1758)

4CC45272-1102-5FF8-9FF2-8A70EB09D3E5

##### Materials

- **Type status:** Other material. **Location:** county: Sulyamaniyah; locality: Qlyasan; verbatimCoordinates: 35°34'41"N, 45°22'01"E

#### Hipparchia fatua

(Freyer, 1845)

E4B8572F-03BA-5B1E-845E-2677EBA8482C

##### Materials

- **Type status:** Other material. **Location:** county: Sulyamaniyah; locality: Hawary Shar Park; verbatimCoordinates: 35°36'41"N, 45°25'48"E

#### Hipparchia parisatis

(Kollar, [1849])

D1107299-105A-561C-8E3A-F848026D1634

##### Materials

- **Type status:** Other material. **Location:** county: Dukan; locality: Zêwê (Piramagroon Mount.); verbatimCoordinates: 35°45'41"N, 45°14'17"E
- **Type status:** Other material. **Location:** county: Halabja; locality: Sargat Village; verbatimCoordinates: 35°17'34"N, 46°06'18"E
- **Type status:** Other material. **Location:** county: Qareh Dagh; locality: Qareh Dagh Mount.; verbatimCoordinates: 35°14'27"N, 45°22'12"E

#### Hipparchia pellucida

(Stauder, 1924)

B5367D38-EE07-56FF-8B59-4633EF0BE996

##### Materials

- **Type status:** Other material. **Location:** county: Sulyamaniyah; locality: Hawary Shar Park; verbatimCoordinates: 35°36'41"N, 45°25'48"E

#### Hipparchia syriaca

(Staudinger, 1871)

474F33F6-96CA-51D0-8C56-ABC960D2C246

##### Materials

- **Type status:** Other material. **Location:** county: Chuarta; locality: Upper Dêrê Village; verbatimCoordinates: 35°56'08"N, 44°57'38"E
- **Type status:** Other material. **Location:** county: Mawat; locality: Galala Village; verbatimCoordinates: 35°53'58"N, 45°19'51"E
- **Type status:** Other material. **Location:** county: Mawat; locality: Mawat; verbatimCoordinates: 35°53'10"N, 45°23'59"E
- **Type status:** Other material. **Location:** county: Chuarta; locality: Little Barê Village; verbatimCoordinates: 35°53'02"N, 45°40'07"E
- **Type status:** Other material. **Location:** county: Dukan; locality: Zêwê (Piramagroon Mount.); verbatimCoordinates: 35°45'41"N, 45°14'17"E
- **Type status:** Other material. **Location:** county: Penjwen; locality: Penjwen; verbatimCoordinates: 35°36'09"N, 45°57'48"E
- **Type status:** Other material. **Location:** county: Halabja; locality: Sargat Village; verbatimCoordinates: 35°17'34"N, 46°06'18"E
- **Type status:** Other material. **Location:** county: Qareh Dagh; locality: Qareh Dagh Mount.; verbatimCoordinates: 35°14'27"N, 45°22'12"E
- **Type status:** Other material. **Location:** county: Halabja; locality: Byara; verbatimCoordinates: 35°13'47"N, 46°07'13"E

#### Hyponephele lupina

(Costa, 1836)

C31719C5-CCE6-5E7D-BCC5-458941AC7CEA

##### Materials

- **Type status:** Other material. **Location:** county: Dukan; locality: Zêwê (Piramagroon Mount.); verbatimCoordinates: 35°45'41"N, 45°14'17"E

#### Hyponephele lycaon

(Rottemburg, 1775)

5C05DEC3-AA00-5A31-A986-33FA6E5FC134

##### Materials

- **Type status:** Other material. **Location:** county: Chuarta; locality: Upper Dêrê Village; verbatimCoordinates: 35°56'08"N, 44°57'38"E

#### Hyponephele wagneri

(Herrich-Schäffer, [1846])

CC5D312E-F3EA-50A4-BFFC-EA273396B719

##### Materials

- **Type status:** Other material. **Location:** county: Dukan; locality: Zêwê (Piramagroon Mount.); verbatimCoordinates: 35°45'41"N, 45°14'17"E

#### Junonia orithya

(Linnaeus, 1758)

B731D0AB-4C51-56B0-B9FB-043B23CB260F

##### Materials

- **Type status:** Other material. **Location:** county: Sulyamaniyah; locality: Hawary Shar Park; verbatimCoordinates: 35°36'41"N, 45°25'48"E
- **Type status:** Other material. **Location:** county: Sulyamaniyah; locality: Azady Park; verbatimCoordinates: 35°34'02"N, 45°25'51"E

#### Lasiommata megera

(Linnaeus, 1767)

C3FC9E64-D6C8-5C0A-A471-FF616FD731E9

##### Materials

- **Type status:** Other material. **Location:** county: Mawat; locality: Galala Village; verbatimCoordinates: 35°53'58"N, 45°19'51"E
- **Type status:** Other material. **Location:** county: Sulyamaniyah; locality: Qlyasan; verbatimCoordinates: 35°34'41"N, 45°22'01"E
- **Type status:** Other material. **Location:** county: Bakrajo; locality: Hazarmerd; verbatimCoordinates: 35°29'56"N, 45°18'54"E

#### Limenitis reducta

Staudinger, 1901

63FA97F4-D7C3-5149-B48F-C10F433390D2

##### Materials

- **Type status:** Other material. **Location:** county: Chuarta; locality: Upper Dêrê Village; verbatimCoordinates: 35°56'08"N, 44°57'38"E
- **Type status:** Other material. **Location:** county: Mawat; locality: Galala Village; verbatimCoordinates: 35°53'58"N, 45°19'51"E
- **Type status:** Other material. **Location:** county: Mawat; locality: Mawat; verbatimCoordinates: 35°53'10"N, 45°23'59"E
- **Type status:** Other material. **Location:** county: Chuarta; locality: Little Barê Village; verbatimCoordinates: 35°53'02"N, 45°40'07"E
- **Type status:** Other material. **Location:** county: Penjwen; locality: Sya Gwez Village; verbatimCoordinates: 35°48'37"N, 45°47'33"E
- **Type status:** Other material. **Location:** county: Dukan; locality: Zêwê (Piramagroon Mount.); verbatimCoordinates: 35°45'41"N, 45°14'17"E
- **Type status:** Other material. **Location:** county: Halabja; locality: Sargat Village; verbatimCoordinates: 35°17'34"N, 46°06'18"E

#### Maniola jurtina

(Linnaeus, 1758)

9D457FD5-DF32-5383-8AFE-E9E788DD78E6

##### Materials

- **Type status:** Other material. **Location:** county: Chuarta; locality: Upper Dêrê Village; verbatimCoordinates: 35°56'08"N, 44°57'38"E
- **Type status:** Other material. **Location:** county: Dukan; locality: Sargalw (Bargalw); verbatimCoordinates: 35°52'44"N, 45°09'49"E
- **Type status:** Other material. **Location:** county: Bakrajo; locality: Kany Pan; verbatimCoordinates: 35°33'03"N, 45°18'00"E

#### Maniola telmessia

(Zeller, 1847)

C5900C19-FFD3-57B6-9CEC-D4D1D909DB0C

##### Materials

- **Type status:** Other material. **Location:** county: Pishdar; locality: Shênê Village; verbatimCoordinates: 36°17'00"N, 45°16'01"E
- **Type status:** Other material. **Location:** county: Chuarta; locality: Upper Dêrê Village; verbatimCoordinates: 35°56'08"N, 44°57'38"E
- **Type status:** Other material. **Location:** county: Mawat; locality: Galala Village; verbatimCoordinates: 35°53'58"N, 45°19'51"E
- **Type status:** Other material. **Location:** county: Mawat; locality: Mawat; verbatimCoordinates: 35°53'10"N, 45°23'59"E
- **Type status:** Other material. **Location:** county: Penjwen; locality: Sya Gwez Village; verbatimCoordinates: 35°48'37"N, 45°47'33"E

#### Melanargia hylata

(Ménétriés, 1832)

E7B37E1F-366C-53E7-BCB0-08DA2A29A21A

##### Materials

- **Type status:** Other material. **Location:** county: Mawat; locality: Galala Village; verbatimCoordinates: 35°53'58"N, 45°19'51"E
- **Type status:** Other material. **Location:** county: Mawat; locality: Mawat; verbatimCoordinates: 35°53'10"N, 45°23'59"E
- **Type status:** Other material. **Location:** county: Dukan; locality: Zêwê (Piramagroon Mount.); verbatimCoordinates: 35°45'41"N, 45°14'17"E

#### Melanargia larissa

(Geyer, [1828])

2FE3B218-B836-58DE-AC69-F6FF9BD36AC7

##### Materials

- **Type status:** Other material. **Location:** county: Sulyamaniyah; locality: Hawary Shar Park; verbatimCoordinates: 35°36'41"N, 45°25'48"E
- **Type status:** Other material. **Location:** county: Bakrajo; locality: Hazarmerd; verbatimCoordinates: 35°29'56"N, 45°18'54"E
- **Type status:** Other material. **Location:** county: Halabja; locality: Sargat Village; verbatimCoordinates: 35°17'34"N, 46°06'18"E

#### Melitaea arduinna

(Esper, 1783)

A301A2D9-32C6-5C41-A0CE-53D3750AE649

##### Materials

- **Type status:** Other material. **Location:** county: Bakrajo; locality: Hazarmerd; verbatimCoordinates: 35°29'56"N, 45°18'54"E

#### Melitaea cinxia

(Linnaeus, 1758)

9A906B5D-ED7F-5C4A-BCFC-200E189F67B2

##### Materials

- **Type status:** Other material. **Location:** county: Qareh Dagh; locality: Qareh Dagh Mount.; verbatimCoordinates: 35°14'27"N, 45°22'12"E

#### Melitaea didyma

(Esper, 1778)

FEA1792F-B3E6-5BE8-ACEC-54F5F59AD138

##### Materials

- **Type status:** Other material. **Location:** county: Chuarta; locality: Upper Dêrê Village; verbatimCoordinates: 35°56'08"N, 44°57'38"E

#### Melitaea gina

Higgins, 1941

D77F70E7-3BCC-50B4-96B3-1E9E405220A9

##### Materials

- **Type status:** Other material. **Location:** county: Pishdar; locality: Shênê Village; verbatimCoordinates: 36°17'00"N, 45°16'01"E

#### Nymphalis polychloros

(Linnaeus, 1758)

B1C66EF6-A8B2-5112-908D-7CA350F6F258

##### Materials

- **Type status:** Other material. **Location:** county: Mawat; locality: Mokaba; verbatimCoordinates: 35°45'26"N, 45°25'41"E

#### Pararge aegeria

(Linnaeus, 1758)

BA2314E2-18E7-5ECB-9901-C7D927C3B360

##### Materials

- **Type status:** Other material. **Location:** county: Ranya; locality: Sarkapkan; verbatimCoordinates: 36°21'04"N, 44°46'24"E
- **Type status:** Other material. **Location:** county: Pishdar; locality: Shênê Village; verbatimCoordinates: 36°17'00"N, 45°16'01"E
- **Type status:** Other material. **Location:** county: Mawat; locality: Mawat; verbatimCoordinates: 35°53'10"N, 45°23'59"E
- **Type status:** Other material. **Location:** county: Dukan; locality: Sargalw (Bargalw); verbatimCoordinates: 35°52'44"N, 45°09'49"E
- **Type status:** Other material. **Location:** county: Penjwen; locality: Sya Gwez Village; verbatimCoordinates: 35°48'37"N, 45°47'33"E
- **Type status:** Other material. **Location:** county: Penjwen; locality: Penjwen; verbatimCoordinates: 35°36'09"N, 45°57'48"E
- **Type status:** Other material. **Location:** county: Halabja; locality: Sargat Village; verbatimCoordinates: 35°17'34"N, 46°06'18"E
- **Type status:** Other material. **Location:** county: Halabja; locality: Byara; verbatimCoordinates: 35°13'47"N, 46°07'13"E

#### Pararge climene

(Esper, 1783)

E089A69D-8DA2-5638-A4E9-AA8245939297

##### Materials

- **Type status:** Other material. **Location:** county: Dukan; locality: Zêwê (Piramagroon Mount.); verbatimCoordinates: 35°45'41"N, 45°14'17"E

#### Pararge roxelana

(Cramer, [1777])

E042A080-CA6C-59AF-9ABD-B82954F82FDF

##### Materials

- **Type status:** Other material. **Location:** county: Dukan; locality: Sargalw (Bargalw); verbatimCoordinates: 35°52'44"N, 45°09'49"E
- **Type status:** Other material. **Location:** county: Qareh Dagh; locality: Qareh Dagh Mount.; verbatimCoordinates: 35°14'27"N, 45°22'12"E

#### Polygonia c-album

(Linnaeus, 1758)

5EC545B3-0B9A-53ED-A5E3-AECA5596B004

##### Materials

- **Type status:** Other material. **Location:** county: Mawat; locality: Mawat; verbatimCoordinates: 35°53'10"N, 45°23'59"E

#### * Pseudochazara mamurra

(Herrich-Schäffer, [1846])

75F215EC-866A-56D4-B6EB-F4B1DCD65E9A

##### Materials

- **Type status:** Other material. **Occurrence:** recordedBy: F. A. Khudhur; sex: 1 female; **Location:** county: Mawat; locality: Galala Village; verbatimCoordinates: 35°53'58"N, 45°19'51"E; **Identification:** identifiedBy: Farhad A. Khudhur; identificationReferences: Eckweiler 2012, Tshikolovets et al. 2014 & Tikhonov et al. 2020; **Event:** eventDate: 11-Jun-20; **Record Level:** basisOfRecord: PreservedSpecimen
- **Type status:** Other material. **Occurrence:** recordedBy: F. A. Khudhur; sex: 1 male; **Location:** county: Dukan; locality: Zêwê (Piramagroon Mount.); verbatimCoordinates: 35°45'41"N, 45°14'17"E; **Identification:** identifiedBy: Farhad A. Khudhur; identificationReferences: Eckweiler 2012, Tshikolovets et al. 2014 & Tikhonov et al. 2020; **Event:** eventDate: 6-Jun-20; **Record Level:** basisOfRecord: PreservedSpecimen

##### Notes

First record for Iraq.

#### Pseudochazara pelopea

(Klug, 1832)

109B9937-BFAA-51A4-BE90-4451C747E842

##### Materials

- **Type status:** Other material. **Location:** county: Chuarta; locality: Upper Dêrê Village; verbatimCoordinates: 35°56'08"N, 44°57'38"E
- **Type status:** Other material. **Location:** county: Dukan; locality: Zêwê (Piramagroon Mount.); verbatimCoordinates: 35°45'41"N, 45°14'17"E

#### Pseudochazara thelephassa

(Geyer, [1827])

52046FE5-7042-5DE8-9264-E0C0038125AF

##### Materials

- **Type status:** Other material. **Location:** county: Dukan; locality: Zêwê (Piramagroon Mount.); verbatimCoordinates: 35°45'41"N, 45°14'17"E
- **Type status:** Other material. **Location:** county: Qareh Dagh; locality: Qareh Dagh Mount.; verbatimCoordinates: 35°14'27"N, 45°22'12"E

#### Vanessa atalanta

(Linnaeus, 1758)

2D94AB28-2EC8-51AE-8DB2-585A5870637A

##### Materials

- **Type status:** Other material. **Location:** county: Dukan; locality: Upper Dukan; verbatimCoordinates: 35°56'59"N, 44°57'38"E
- **Type status:** Other material. **Location:** county: Sulyamaniyah; locality: Hawary Shar Park; verbatimCoordinates: 35°36'41"N, 45°25'48"E
- **Type status:** Other material. **Location:** county: Sulyamaniyah; locality: Azady Park; verbatimCoordinates: 35°34'02"N, 45°25'51"E
- **Type status:** Other material. **Location:** county: Bakrajo; locality: Hazarmerd; verbatimCoordinates: 35°29'56"N, 45°18'54"E

#### Vanessa cardui

(Linnaeus, 1758)

773664AD-8BEB-5940-A347-049425582107

##### Materials

- **Type status:** Other material. **Location:** county: Ranya; locality: Sarkapkan; verbatimCoordinates: 36°21'04"N, 44°46'24"E
- **Type status:** Other material. **Location:** county: Pishdar; locality: Shênê Village; verbatimCoordinates: 36°17'00"N, 45°16'01"E
- **Type status:** Other material. **Location:** county: Dukan; locality: Qamchukha Village; verbatimCoordinates: 35°53'51"N, 45°00'51"E
- **Type status:** Other material. **Location:** county: Chamchamal; locality: Goptapa Village; verbatimCoordinates: 35°51'00"N, 44°50'07"E
- **Type status:** Other material. **Location:** county: Dukan; locality: Chami Razan Valley; verbatimCoordinates: 35°48'03"N, 44°58'38"E
- **Type status:** Other material. **Location:** county: Dukan; locality: Zêwê (Piramagroon Mount.); verbatimCoordinates: 35°45'41"N, 45°14'17"E
- **Type status:** Other material. **Location:** county: Mawat; locality: Mokaba; verbatimCoordinates: 35°45'26"N, 45°25'41"E
- **Type status:** Other material. **Location:** county: Sulyamaniyah; locality: Hawary Shar Park; verbatimCoordinates: 35°36'41"N, 45°25'48"E
- **Type status:** Other material. **Location:** county: Sulyamaniyah; locality: Azady Park; verbatimCoordinates: 35°34'02"N, 45°25'51"E
- **Type status:** Other material. **Location:** county: Barzinja; locality: Basak Village; verbatimCoordinates: 35°33'30"N, 45°42'57"E
- **Type status:** Other material. **Location:** county: Bakrajo; locality: Kany Pan; verbatimCoordinates: 35°33'03"N, 45°18'00"E
- **Type status:** Other material. **Location:** county: Bazyan; locality: Dêlêzha; verbatimCoordinates: 35°27'36"N, 45°11'26"E
- **Type status:** Other material. **Location:** county: Said Sadiq; locality: Nawê Village; verbatimCoordinates: 35°24'42"N, 45°57'59"E
- **Type status:** Other material. **Location:** county: Darbandikhan; locality: Sartak; verbatimCoordinates: 34°56'45"N, 45°46'32"E

## Discussion

This study is first intensive faunistic study of butterfly in Sulaymaniyah Province. The diverse topography and ecosystems in Sulaymaniyah Province contributes to the richness in its biodiversity (Ararat 2009). In particular, the area has a mixed long border and extended geography with three areas of rich biodiversity: the Zagrosian Provinces of Iran including West Azarbayjan, Kordestan and Kermanshah (Haidari 2012, Kazemi and Hossienzadeh 2020). The results of this study reflect this biodiversity richness. In addition to the 154 species of butterflies that have been documented previously in Iraq (Wiltshire 1957, Higgins 1958, Igarashi 1971, Othman et al. 2018, Said et al. 2018, Yakovlev 2018, Khudhur 2021), 95 species were founded in Sulaymaniyah Province during these investigations. Moreover, this study adds eight new species previously unrecorded to the butterfly fauna of Iraq. Three of these newly-found species within Iraq belong to Pieridae; *Pieriskrueperi*, *Gonepteryxrhamni* and *Coliaserate*. The five others were two skippers, *Carcharodusstauderi* and *Thymelicushyrax*; two Nymphalids *Brenthismofidii* and *Pseudochazaramamurra* and a lycaenid butterfly *Polyommatusthersites*. All of these new records were predicted to be found here, since Sulaymaniyah is located near to the known distribution range of these species (Wiltshire 1957, Eckweiler 2012, Tshikolovets et al. 2014, Tikhonov et al. 2020). Several works of literature (Kemal and Koçak 2011, Bozano et al. 2016, Kemal and Koçak 2018) indicated distribution of these mentioned species in Iraq, based on the country position within the range of the Westren Palaearctic Region, while these indications were not supported by referencing to specific preserved specimens from Iraq. More new records of butterflies are likely to be found in this region and neighbouring areas in the future studies. Amongst the visited localities of this study, Piramagroon Mountain (Zêwê Village, locality no.15) was unique and had the richest diversity regarding butterflies with the highest number of species recorded, including the three new records *Carcharodusstauderi*, *Gonepteryxrhamni* and *Pseudochazaramamurra*. Furthermore, several other rarely found species were collected from Piramagroon Mountain, including *Favoniusquercus*, *Satyriumgerhardi*, *Hyponephelewagneri*, *Hyponephelelupine*, *Parargeclimene* and *Erynnismarloyi*. Therefore, this mountain deserves further investigations and will be the next focus for our future work.

## Figures and Tables

**Table 1.** Geographical information of investigated localities.

Loc. No.	Locality	Latitude / Longitude	Elevation (m)	Visiting Date
1	Sarkapkan	36°21'04"N, 44°46'24"E	791	07.05.2016
				20.09.2018
2	Shênê	36°17'00"N, 45°16'01"E	1290	12.08.2020
3	Shanakhsê	35°58'37"N, 45°31'11"E	1060	11.07.2020
4	Dukan	35°56'59"N, 44°57'38"E	515	18.11.2016
				15.05.2018
5	Upper Dêrê	35°56'08"N, 44°57'38"E	1600	21.06.2020
				01.07.2020
6	Galala	35°53'58"N, 45°19'51"E	1170	11.06.2020
7	Qamchukha	35°53'51"N, 45°00'51"E	712	06.09.2016
8	Mawat	35°53'10"N, 45°23'59"E	786	30.08.2016
				12.04.2020
				21.08.2020
9	Little Barê	35°53'02"N, 45°40'07"E	1500	06.09.2020
10	Sargalw	35°52'44"N, 45°09'49"E	930	17.05.2020
11	Goptapa	35°51'00"N, 44°50'07"E	376	02.05.2017
12	Sya Gwez	35°48'37"N, 45°47'33"E	1500	24.08.2016
13	Chami Razan	35°48'03"N, 44°58'38"E	586	12.04.2016
				06.09.2016
14	Balkha	35°47'09"N, 45°22'47"E	855	16.08.2017
15	Gollê	35°46'31"N, 45°49'54"E	1117	29.06.2020
16	Zêwê (Piramagroon Mountain)	35°45'41"N, 45°14'17"E	1720	15.08.2016
				12.09.2018
				06.06.2020
				10.08.2020
				04.05.2021
17	Mokaba	35°45'26"N, 45°25'41"E	841	25.04.2018
				09.04.2021
18	Qaiwan	35°42'01"N, 45°25'03"E	1230	21.05.2016
				13.05.2017
19	Hawary Shar Park	35°36'41"N, 45°25'48"E	919	28.08.2016
				23.10.2016
				21.07.2018
				20.11.2018
				29.05.2020
				06.04.2021
20	Penjwin	35°36'09"N, 45°57'48"E	1400	18.04.2019
21	Goyzha	35°34'57"N, 45°28'09"E	1070	27.09.2016
22	Qlyasan	35°34'41"N, 45°22'01"E	756	24.09.2016
				09.11.2016
				29.03.2017
				09.06.2017
				25.06.2019
				27.06.2020
23	Azady Park	35°34'02"N, 45°25'51"E	845	17.08.2016
				29.10.2016
				14.04.2021
24	Basak	35°33'30"N, 45°42'57"E	1260	05.05.2016
25	Kani Pan	35°33'03"N, 45°18'00"E	714	05.10.2016
				12.10.2016
26	Hazarmerd	35°29'56"N, 45°18'54"E	958	03.05.2016
				21.04.2017
				15.04.2019
				05.05.2020
				14.05.2020
				06.08.2020
				13.10.2020
				15.04.2021
27	Dêlêzha	35°27'36"N, 45°11'26"E	695	26.04.2016
				01.09.2020
28	Nawê	35°24'42"N, 45°57'59"E	656	27.10.2017
29	Zalm	35°18'53"N, 46°05'07"E	843	11.05.2020
30	Sargat	35°17'34"N, 46°06'18"E	1050	16.06.2020
31	Qareh Dagh	35°14'27"N, 45°22'12"E	1310	06,05,2017
				30.05.2020
				01.09.2020
				12.09.2020
32	Byara	35°13'47"N, 46°07'13"E	1120	22.09.2016
				07.07.2017
				14.09.2019
33	Sartak	34°56'45"N, 45°46'32"E	1270	20.05.2017
				19.03.2021
34	Awa Khwery	34°53'30"N, 45°33'29"E	311	16.08.2019
